# Editorial: Tumor microenvironment and hematological malignancies: new evidences and new questions

**DOI:** 10.3389/fimmu.2024.1407981

**Published:** 2024-04-16

**Authors:** Stefania Fiorcari, Paolo Strati, Elisabetta Dondi

**Affiliations:** ^1^ Department of Oncology and Hematology, Azienda Ospedaliero Universitaria di Modena, Modena, Italy; ^2^ Department of Lymphoma and Myeloma & Department of Translational Molecular Pathology The University of Texas MD (UT MD) Anderson Cancer Center, Houston, TX, United States; ^3^ U978 Institut National de la Santé et de la Recherche Médicale/Université Sorbonne Paris Nord, Labex INFLAMEX, Bobigny, France

**Keywords:** tumor microenvironment, hematological malignancies, immune escape, homeostasis, niches, signaling

The tumor microenvironment (TME) plays an essential role in the development of hemopoietic malignancies. Within the proliferation niches of lymph nodes, bone marrow, and secondary lymphoid organs, different cell types provide survival and growth factors to malignant cells. These subpopulations, including nonhematopoietic stromal cells, extra-cellular matrix, lymphocytes, and myeloid cells, present multiple phenotypic and functional alterations. Dynamic crosstalk between hematopoietic tumor cells and the TME actively shapes a tumor-supporting niche that significantly impact tumor progression by leading to immune escape mechanisms and subsequent response to treatment. Moreover, an altered availability of metabolites might contribute to a dysregulated immunological status and to detrimental functions of the microenvironment cells. The essential functions of the niche in supporting homeostasis in bone marrow and secondary lymphoid organs are thus turned in a detrimental support to cancer development (1–4).

The translational relevance of targeting TME is of high importance. Increasing evidences about the critical role of a deviant microenvironment in the initiation and maintenance of pathological conditions suggest to interfere with its deleterious protective functions. Therapeutic approaches to restore protective antitumor immunity through interference with the recruitment of myeloid cells, repolarization of immune cell subsets and inhibition of tumor promoting signals are promising strategies (5, 6). Understanding the series of metabolic changes and functional plasticity experienced by TME cells will help to identify new targets for tumor immunotherapy and develop more effective tumor treatment strategies.

The Research Topic: “*Tumor microenvironment and hematological malignancies: new evidences and new questions”* comprises three reviews and three original research articles describing the roles of different types of microenvironmental cells as well as the impact of metabolic changes. A particular interest is given to the possibilities of targeting TME in tumor immunotherapy.

Historical data analyzing the impact of the immune microenvironment on B-cell lymphoma (BCL) have been focused on T-cells, identifying senescence, exhaustion and immune depletion as crucial determinants of dismal outcome (7).

The impact of tumor-infiltrating lymphocytes (TILs) including normal B cells, T cells and natural-killer (NK) cells on the clinical outcomes of diffuse B-cell lymphoma (DLBL) patients, treated with standard chemoimmunotherapy is highlighted in the research article by Yu et al. In this paper, they studied the tumor immune microenvironment (TIME) by flow cytometry in fresh lymphoma tissue fragment isolated from a cohort of DCBL patients. Particularly they showed that higher percentages of normal B cells among total B cells (or high ratios of normal B cells to abnormal B cells) and high percentages of NK cells among all viable cells correlated with significantly better outcomes in patients with DLBCL. On the basis of clinical and flow cytometry factors, they proposed a prognostic model which divided the DLBCL cohort into two equal groups with remarkable differences in patient survival and treatment response. An interesting point in their discussion concerned the differences observed in TIME data between flow cytometry and IHC or genetic analysis in DLBCL. These observations suggested potential caveats of single-cell data requiring tissue disaggregation and underly the need of complementary approaches to fully characterize the TIME.

Recent data have however suggested that also myeloid cells may be impacting sensitivity and resistance to treatment in different subtypes of BCLs (8). In this Research Topic, Dhar et al. show that circulating monocytic myeloid-derived suppressor cells could mediate resistance to multiple chemotherapy agents typically utilized for the treatment of BCL, both in in-vitro and in-vivo models. This important finding rises the possibility of utilizing myeloid immune checkpoints to enhance the activity of chemotherapy for this patient population, beyond the already widely investigated CD47 or “do-not-eat-me” pathway (9). This could however be further complicated by the very impact chemotherapy may have on myeloid cells. In fact, multiple studies have shown that several of the agents currently utilized for the treatment of BCL may affect both the number and phenotype of tumor associated macrophages and MDSCs (10). Of interest, myeloid cells may affect not only response to standard chemotherapy, but also toxicities associated with the use of cellular therapy in BCL. While the introduction of chimeric antigen receptor (CAR) T-cell therapy (CART) has revolutionized the treatment and outcomes of patients with relapsed or refractory BCL, it has not come without a cost (11). Beyond the classical and fully understood side effects, such as cytokine release syndrome and immune cell associated neurotoxicity syndrome, prolonged cytopenia has increasingly become a clinically unmet need in the field. Recent data have demonstrated in fact that the latter represents the main factor excluding patients with relapsed BCL from access to potentially life-saving clinical trials (12). While pre-treatment laboratory values can help predict who is more likely to develop persistent severe cytopenia after CART, its biological mechanisms seems to go beyond the myelosuppression associated with the use of lymphodepleting chemotherapy, and rather be more closely related to CAR T-cells activity (13, 14) In this Research Topic, Sun et al. review recent translational findings, pointing toward interferon-γ-mediated impairment of hematopoietic stem cells as the key biological mechanism for this phenomenon. As more pre-clinical data are generated, agents able to target the myeloid cell-mediated inflammatory abrasion, including eltrombopag and emapalumab, could help address this currently uncurable condition.

Tumor-associated macrophages (TAMs), which are broadly classified as anti-tumor M1 and pro-tumor M2 subtypes, are indeed the most common kind of tumor-infiltrating leucocytes in many malignancies. Notably, the tumor manipulates the TME in such a way that it induces macrophage infiltration and M1 to M2 switching bias to secure its survival. This M2-TAM bias drives not just carcinogenesis via cancer-related inflammatory processes, but also tumor development, invasion, and metastasis. TAMs are also often responsible for the inadequacy of conventional therapies like chemotherapy and radiotherapy to restrain cancer growth and the failure of innovative immunotherapies premised on immune-checkpoint suppression (15, 16).

On these basis, metabolic reprogramming of M2-like TAMs, preventing the recruitment of mononuclear cells or directly deleting M2-like TAMs in tumor tissues have emerged as promising techniques for targeted TAM immunotherapy in solid tumors (17). The review by Khan et al. primarily focused on the origin and functional plasticity of TAMs in response to the tumor microenvironment, in order to understand the series of metabolic and functional alterations experienced. The authors then extensively illustrated the current targeted therapeutic methods, examining the possibilities of targeting TAMs in tumor immunotherapy. They finally suggested that combining TAM-targeted immunotherapy with other tumor treatments could have a more potent anti-tumor effect. This could be especially important in light of the possibility of drug resistance to TAM therapy and of cytotoxic side effects that may be produced by large reduction or overwhelming reversal of TAM.

In AML, bone marrow microenvironment plays a pivotal role for promoting and sustaining leukemogenesis and AML blasts can induce BMSC to differentiate into osteoblasts leading to a more supportive “habitat” (3). In this Research Topic, Tomasoni et al. show that AML cells trigger osteogenic commitment of BMSC leading to a permissive microenvironment to leukemia growth. Notch activation seems to play a pivotal role in the crosstalk between AML cells and BMSC supporting AML progression and protection from drug-induced apoptosis. This finding points up the attention on the possibility to target Notch signaling pathway to disrupt the crosstalk between AML cells and BMSC.

Metabolic niche inside tumor microenvironment is a hallmark of cancer and represents an immunosuppressive environment to be overcome. The metabolism of cancer cells is reprogrammed from that of normal cells. Hypoxia, lack of nutrients (as glucose), acidification, accumulation of lipids, amino acids, lactate allow disease progression (18). Metabolites in the tumor microenvironment may induce dysregulation of gene expression involved in differentiation, proliferation and activation of immune effector cells affecting the epigenetic programs and signal transduction networks (19). In this Research Topic, Chen et al. review the involvement of metabolites in tumor microenvironment, transporters in immune cells and immune cell function. Understanding the modulation and changes in metabolite and immune activity inside the complexity of tumor microenvironment could help to explore a more targeted therapy focused on metabolic profile to allow an effective antitumor immune response.

## Author contributions

ED: Writing – original draft. SF: Writing – original draft. PS: Writing – original draft.
